# Diamond nanophotonics

**DOI:** 10.3762/bjnano.3.100

**Published:** 2012-12-21

**Authors:** Katja Beha, Helmut Fedder, Marco Wolfer, Merle C Becker, Petr Siyushev, Mohammad Jamali, Anton Batalov, Christopher Hinz, Jakob Hees, Lutz Kirste, Harald Obloh, Etienne Gheeraert, Boris Naydenov, Ingmar Jakobi, Florian Dolde, Sébastien Pezzagna, Daniel Twittchen, Matthew Markham, Daniel Dregely, Harald Giessen, Jan Meijer, Fedor Jelezko, Christoph E Nebel, Rudolf Bratschitsch, Alfred Leitenstorfer, Jörg Wrachtrup

**Affiliations:** 1 Department of Physics and Center for Applied Photonics, Konstanz, Germany; 2 3. Physikalisches Institut and Scope Research Centre University of Stuttgart, Stuttgart, Germany; 3 Fraunhofer-Institut für Angewandte Festkörperphysik, Freiburg i. Br., Germany; 4 Institut Neel, CNRS and Université Joseph Fourier, Grenoble, France; 5 Institut für Quantenoptik, Universität Ulm, Ulm, Germany; 6 RUBION, Ruhr-Universität Bochum, Bochum, Germany; 7 Element Six Ltd, Ascot, United Kingdom; 8 4. Physikalisches Institut and Scope Research Centre, Stuttgart, Germany; 9 Institute of Physics, Chemnitz University of Technology, Chemnitz, Germany

**Keywords:** CVD diamond doping, diamond, nanophotonics, NV center, plasmonic resonator, solid immersion lens

## Abstract

We demonstrate the coupling of single color centers in diamond to plasmonic and dielectric photonic structures to realize novel nanophotonic devices. Nanometer spatial control in the creation of single color centers in diamond is achieved by implantation of nitrogen atoms through high-aspect-ratio channels in a mica mask. Enhanced broadband single-photon emission is demonstrated by coupling nitrogen–vacancy centers to plasmonic resonators, such as metallic nanoantennas. Improved photon-collection efficiency and directed emission is demonstrated by solid immersion lenses and micropillar cavities. Thereafter, the coupling of diamond nanocrystals to the guided modes of micropillar resonators is discussed along with experimental results. Finally, we present a gas-phase-doping approach to incorporate color centers based on nickel and tungsten, in situ into diamond using microwave-plasma-enhanced chemical vapor deposition. The fabrication of silicon–vacancy centers in nanodiamonds by microwave-plasma-enhanced chemical vapor deposition is discussed in addition.

## Introduction

Single quantum emitters coupled to plasmonic and dielectric microresonators hold promise for novel photonic devices, such as optical transistors [1], optical quantum memories [2–3] and controlled single-photon sources. Color centers in diamond are well suited quantum emitters with outstanding coherence properties of their electron spin and single-photon operation even at room temperature. However, several key challenges need to be addressed to fully benefit from the above-mentioned properties. First of all, color centers in diamond need to be created in a well-defined way, and new color centers with desired emission and spin properties for quantum optics need to be identified. Both ion implantations as well as doping of diamond during CVD growth are of importance here. Furthermore, future applications rely on color centers with high emission rates. Resonator structures offer an enhanced light out-coupling and an increased spontaneous emission. The remaining challenge here is the coupling of color centers to nanophotonic devices. This is for two reasons: First, most of the emitters are relatively broadband. Therefore, optical resonators must be engineered with a short length, i.e., the mode volume of the resonator should be small. Second, to achieve strong coupling, a single color center must be placed at the maximum of the optical field with high spatial precision. In the present case of broadband plasmonic structures, typically about 10 nm positioning accuracy must be achieved. Herein, the controlled positioning of single color centers in diamond is realized with nanometer spatial precision by ion-beam implantation through nanometer-sized apertures and by fabricating plasmonic structures with hot spots around diamond nanocrystals.

## Results and Discussion

### 1 Creation of single color centers in diamond with nanometer spatial control

An approach that is well suited to create single color centers in diamond with nanometer spatial control is the implantation of nitrogen ions through a mask [4]. At low implantation energies, which generate color centers a few nanometers below the surface, polymer resists with apertures written by electron beam lithography can be used. At higher energies, which create color centers up to a micron deep inside the diamond, suitable masks are thin mica sheets (thickness few micrometers), which contain channels a few nanometers wide. Such channels can be created by bombardment with high-energy heavy ions [4]. These mica masks provide the required thickness to stop those ions that do not enter the apertures, and at the same time provide the required high aspect ratio of the apertures (the channels) to ensure a narrow width of the implantation beam. Both methods are complementary to provide the controlled creation of color centers with low and high implantation energy, respectively. In the following, the second method shall be discussed. A third approach that is suitable in the context of plasmonic structures is to use nanometer-sized diamond crystals with embedded color centers and fabricate plasmonic structures around them. This approach will be discussed in the subsequent section.

**Figure 1:**
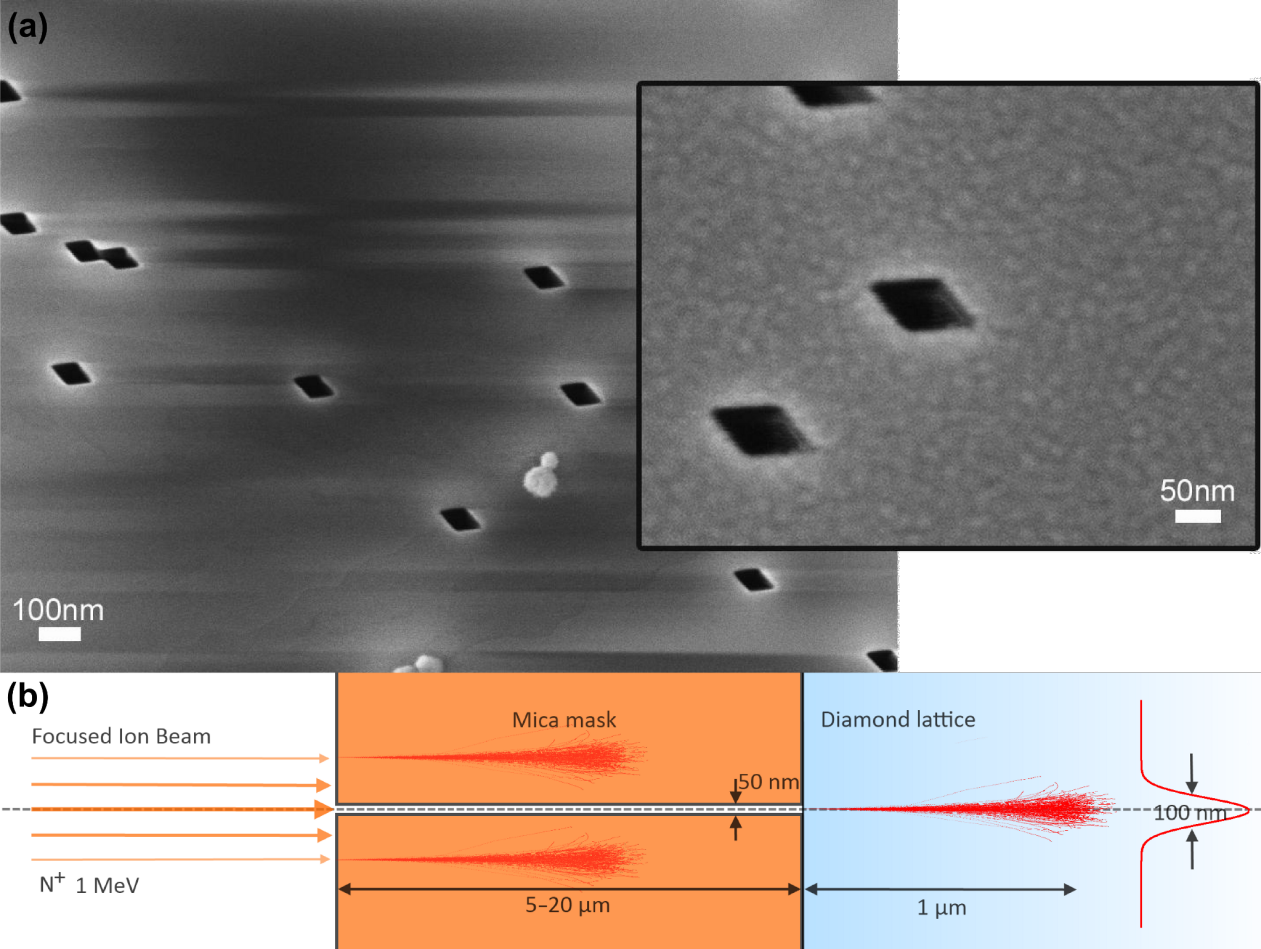
(a) Scanning electron micrograph of a mica mask. High aspect ratio channels were created by bombardment with 1.6 GeV samarium ions. The channels appear as dark parallelograms. The inset shows the dimensions of an individual ion channel. (b) SRIM simulation of the ion implantation process through the mica mask. The thickness of the mica mask is chosen in the range 5–20 μm, such that the nitrogen ions (N^+^) are effectively stopped by the mask. The ions entering the channel create an implanted ion spot with a FWHM of about 100 nm, limited by straggle.

**Figure 2:**
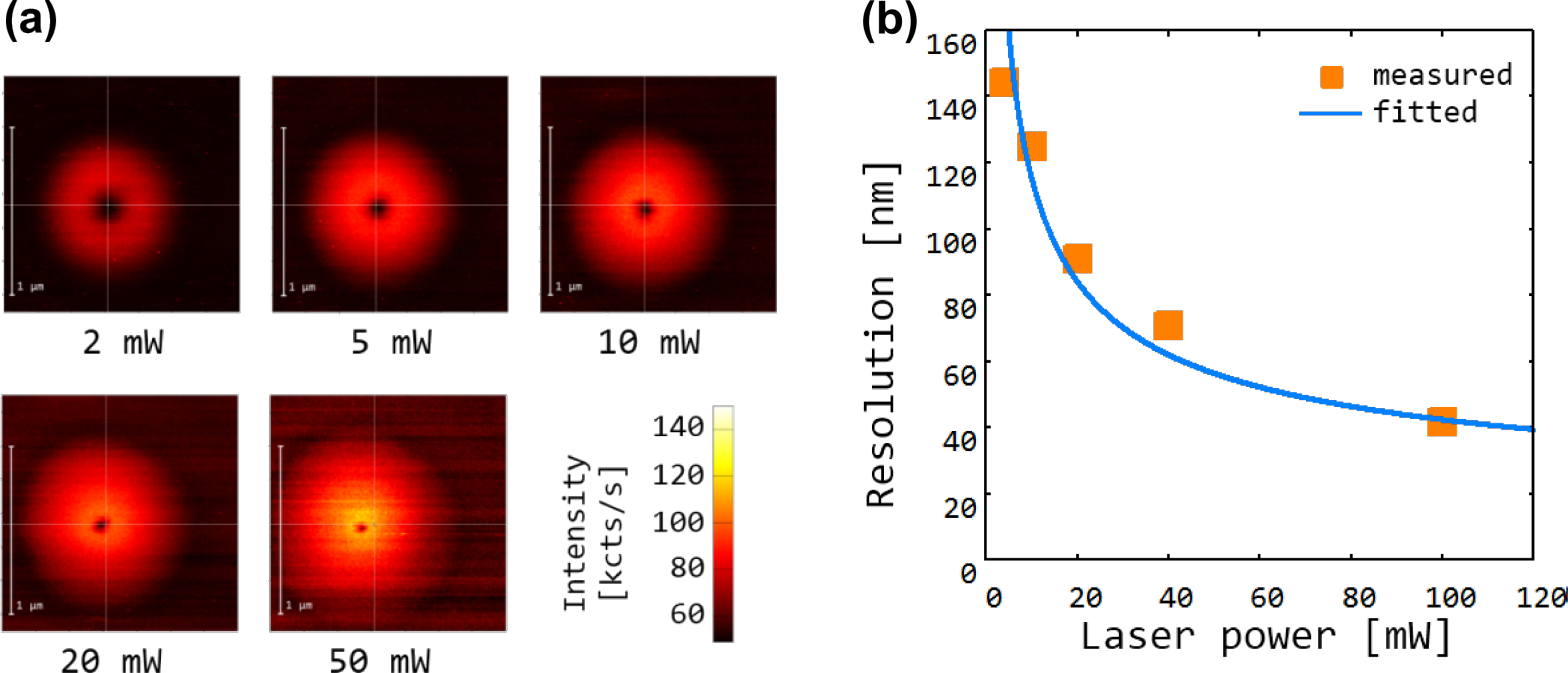
Nonlinear optical microscopy of implanted color centers by using ground-state-depletion microscopy mode. (a) Images of a color center obtained with increasing depletion laser power. (b) Measured optical resolution as a function of laser power. The solid line shows a theoretical fit for the achievable resolution 
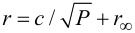
, where c is a proportionality constant, P is the optical power and *r*_∞_ represents the maximum achievable resolution at infinite power, which is determined by technical limitations such as imperfections of the optical mode [6].

Figure 1a shows an electron micrograph of a mica mask. The ion channels have a width of about 50 nm, which is well suited for high-energy implantation with high spatial resolution. The channels have a rhombic cross section that reflects the crystal structure of the mica. The mica mask is then placed on the surface of a diamond. Electrostatic forces ensure good sticking of the mica sheet on the diamond surface. Subsequently, the masked diamond is irradiated with a nitrogen ion (N^+^) beam with an energy of 1 MeV, thereby creating implanted spots with about 100 nm spatial control [4]. Note that with the given high implantation energy, the spatial accuracy of the implantation process is determined entirely by straggle, i.e., the deviation of the ion trajectory inside the diamond crystal, caused by collisions with the lattice atoms. Such implantation processes can be studied in detail with theoretical simulations. A suitable method is scattering calculations. The “Stopping Range of Ions in Matter” (SRIM) package implements such simulations [5]. Figure 1b shows the simulated ion traces achieved with the high-aspect-ratio mica mask. The simulations confirm that the spatial accuracy of the implantation is limited by straggle to about 100 nm. Figure 2a shows a high-resolution optical microscope image of a single implanted color center obtained with nonlinear optical excitation in ground state depletion (GSD) mode [6]. In this imaging mode, the color center is illuminated with a doughnut-shaped beam of high optical intensity. The saturation behavior of the optical transition provides a nonlinear relation between illumination intensity and observed fluorescence, which enables one to overcome the Rayleigh resolution limit of classical optical imaging. The image is generated by scanning the beam in the image plane. The resulting fluorescence image is the doughnut-shaped illumination pattern multiplied by the saturation function of the color center. At low laser power (first image) the fluorescence is proportional to the illuminated light field and the resulting image is equivalent to the illuminating doughnut beam. In this case the central dark spot has a size on the order of the optical wavelength. At higher laser power, the nonlinearity of the saturation function becomes important and the central dark spot shrinks well below the optical wavelength, providing super-resolution optical imaging capability. This technique is suitable to determine the position of an implanted color center with high precision. The accuracy of this imaging method is limited only by the applicable laser power and ultimately by instrument drift of the sample scanning unit. The mica mask implantation and GSD imaging method are suitable to create and characterize one or more color centers in diamond with sub 100 nm spatial control deep inside the diamond crystal (Figure 2b). This deep implantation is of great importance when spin and optical properties must be of the highest quality and well protected from the environment.

### 2 Coupling of nanodiamond quantum emitters to plasmonic resonators

**Figure 3:**
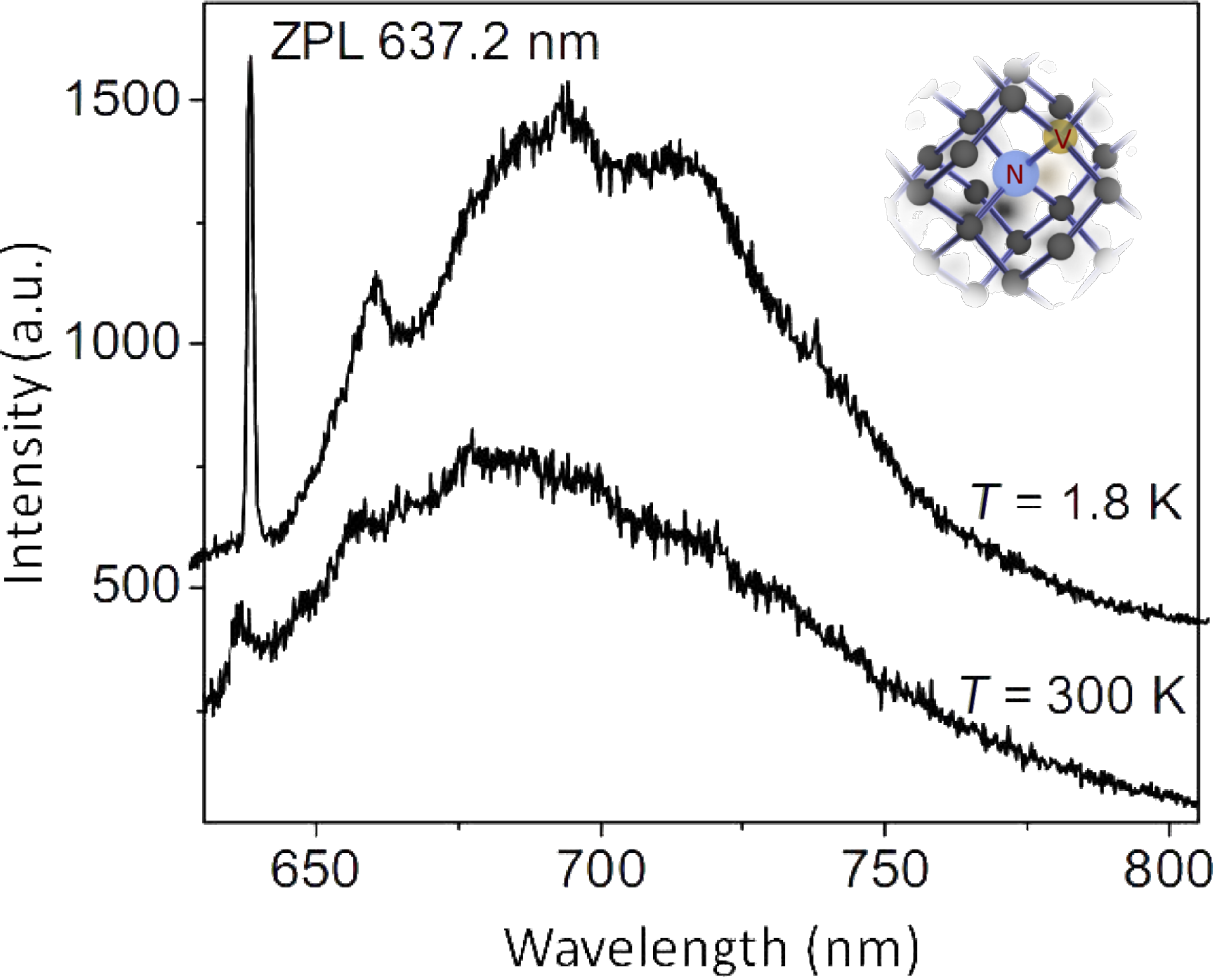
Fluorescence spectra of the nitrogen–vacancy defect in diamond. The upper curve shows the spectrum at liquid-helium temperature, the lower curve shows the spectrum at room temperature. The peak at a wavelength of 637.2 nm corresponds to the resonant optical transition (ZPL). The broad band at longer wavelength corresponds to phonon-broadened emission. The inset shows the molecular structure of the NV center in diamond consisting of a substitutional nitrogen atom with an adjacent vacancy.

**Figure 4:**
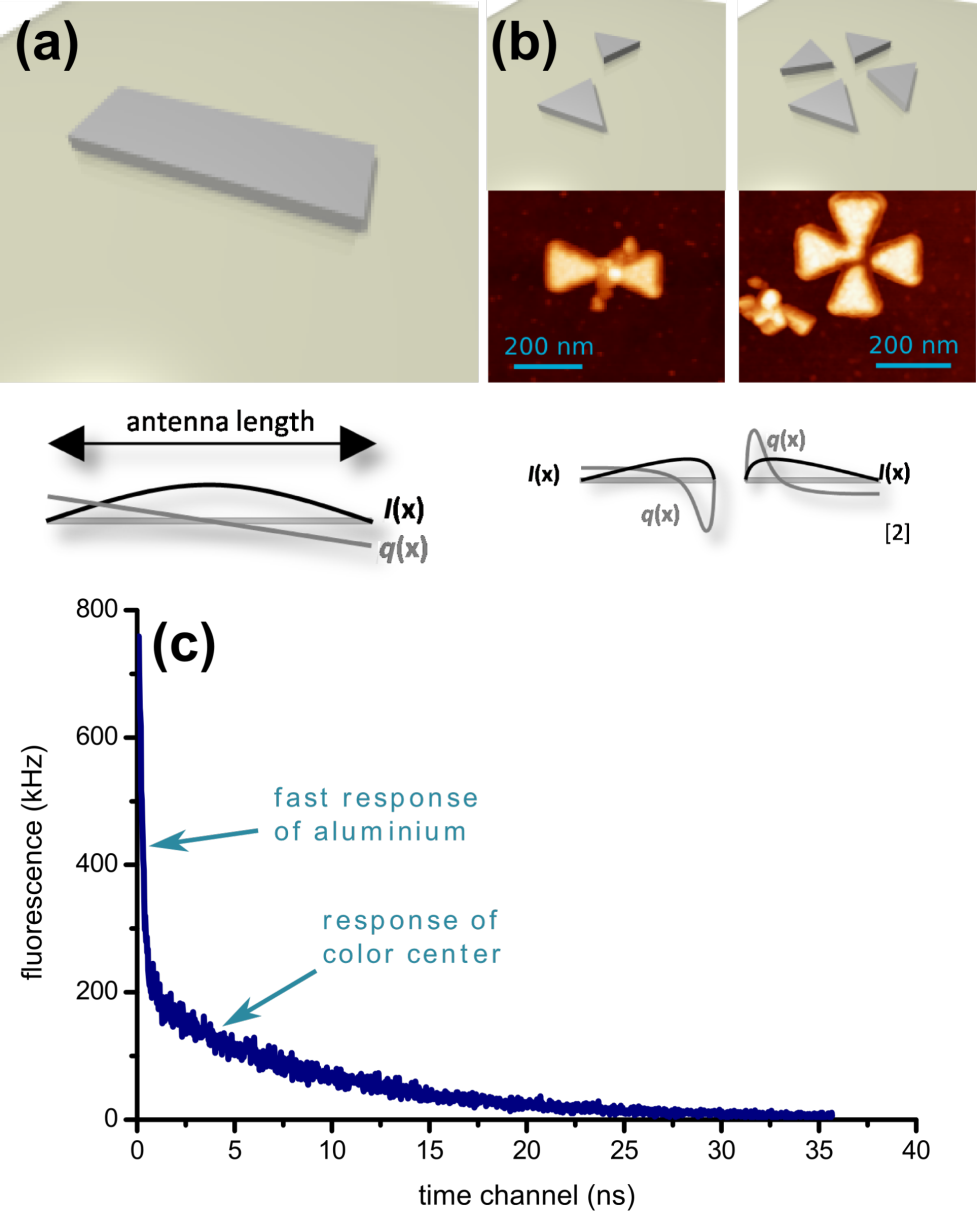
Plasmonic resonator geometries, field *I* and current *q* for (a) half-wave antenna, (b) bow tie and crossed bow tie structure along with AFM images of the devices with embedded nanodiamond color center. (c) Fluorescence lifetime measurement of the coupled color center. A double exponential decay is observed. The initial fast decay is due to aluminium interband transitions and background. The second, slow decay is due to the coupled color center.

At room temperature, most solid state defects have a broadband optical emission spectrum. The resonant optical line width is typically several nanometers wide. Frequently, in addition to the resonant line, a broad emission band is observed that can be up to few 100 nm wide. This broad emission band corresponds to vibrational levels and its strength depends on how much the optical emission couples to lattice vibrations. Figure 3 shows a typical room-temperature optical emission spectrum of the nitrogen–vacancy (NV) color center in diamond. The resonant optical emission appears as a weak peak at a wavelength of 637 nm (zero-phonon line, ZPL), and a broad emission band ranging from about 630 up to 750 nm is observed. In order to couple such broadband quantum emitters to a resonant optical light field a suitable broadband optical resonator is required. To realize a broadband resonator that has at the same time a high finesse, the optical mode volume must be sufficiently small. Plasmonic resonators are well suited to provide such small mode volumes. In this case metals are used rather than dielectrics to confine optical light fields. The negative refractive index of the metal ensures that guided (localized) modes exist, even when the dimensions of the device are much smaller than the optical wavelength. In this way, resonators with mode volumes much smaller than a cubic wavelength can be realized. Such structures are often optical equivalents of corresponding macroscopic electromagnetic antennas. Figure 4 illustrates some of the antennas considered in this study [7–9]. The prototype of a resonant plasmonic antenna is a metallic strip (Figure 4a) with a width and thickness of few tens of nanometers and a length corresponding to half of the optical wavelength. The electric field, *I,* has a maximum in the center of the stripe. Quantum emitters should be placed as close as possible to this location. The coupling between a quantum emitter and the optical field can be enhanced by cutting the antenna in two parts and creating a small (about 10–20 nm) gap in between, analogous to the feed gap of a radio antenna. The quantum emitter is placed in the feed gap, where the optical field is maximal. The field in the gap can be increased by tapering both antenna arms and reshaping their ends at the feed gap with sharp tips. This leads to the so-called “bow tie” antenna structure shown in Figure 4b. An antenna with polarization independent far-field radiation pattern can be created by combining two bow-tie antennas to a crosslike structure as also shown in Figure 4b.

An approach that is suitable to couple solid state quantum emitters to plasmonic resonators is the use of diamond nanocrystals containing photoactive color centers. Such diamond nanocrystals can be as small as 10 nm. By using suitable fabrication steps, plasmonic structures can be fabricated around such crystals with precise spatial control. In this process, first, gold markers are fabricated on a glass substrate by using electron beam lithography. Subsequently, diamond nanocrystals are spin coated onto the substrate. By using a dual atomic force microscope (AFM) and confocal microscopy setup, diamond nanocrystals that contain single color centers are then identified by fluorescence microscopy and second-order photon autocorrelation, and their position relative to the gold markers is measured with nanometer precision by AFM. Finally, plasmonic structures are fabricated around the selected diamond nanocrystals.

The middle panel of Figure 4b shows AFM images of the plasmonic resonators coupled to diamond nanocrystals. A positioning accuracy of about 20 nm is achieved. This ensures that the quantum emitters are located well inside the hot spot of the plasmonic structures. A method that is suitable to verify the coupling of the color centers to the resonators is optical lifetime measurements. Lifetime measurements were performed with supercontinuum pulsed laser excitation with a pulse length of about 50 ps. Figure 4c shows a typical lifetime measurement. A double exponential decay is observed. The initial fast decay is due to fast interband transitions of the metal and background fluorescence. The second slow decay is due to the color center. Table 1 summarizes the observed decay constants. For all resonators, the decay time is reduced by about a factor of four compared to an uncoupled color center. The strongest coupling is observed with the bow-tie resonator.

**Table 1:** Decrease of the fluorescence lifetimes.

resonator	lifetime [ns]

uncoupled	24
wire	6.6
bow tie	3.6
cross	4.2

### 3 Dielectric diamond photonics

**Figure 5:**
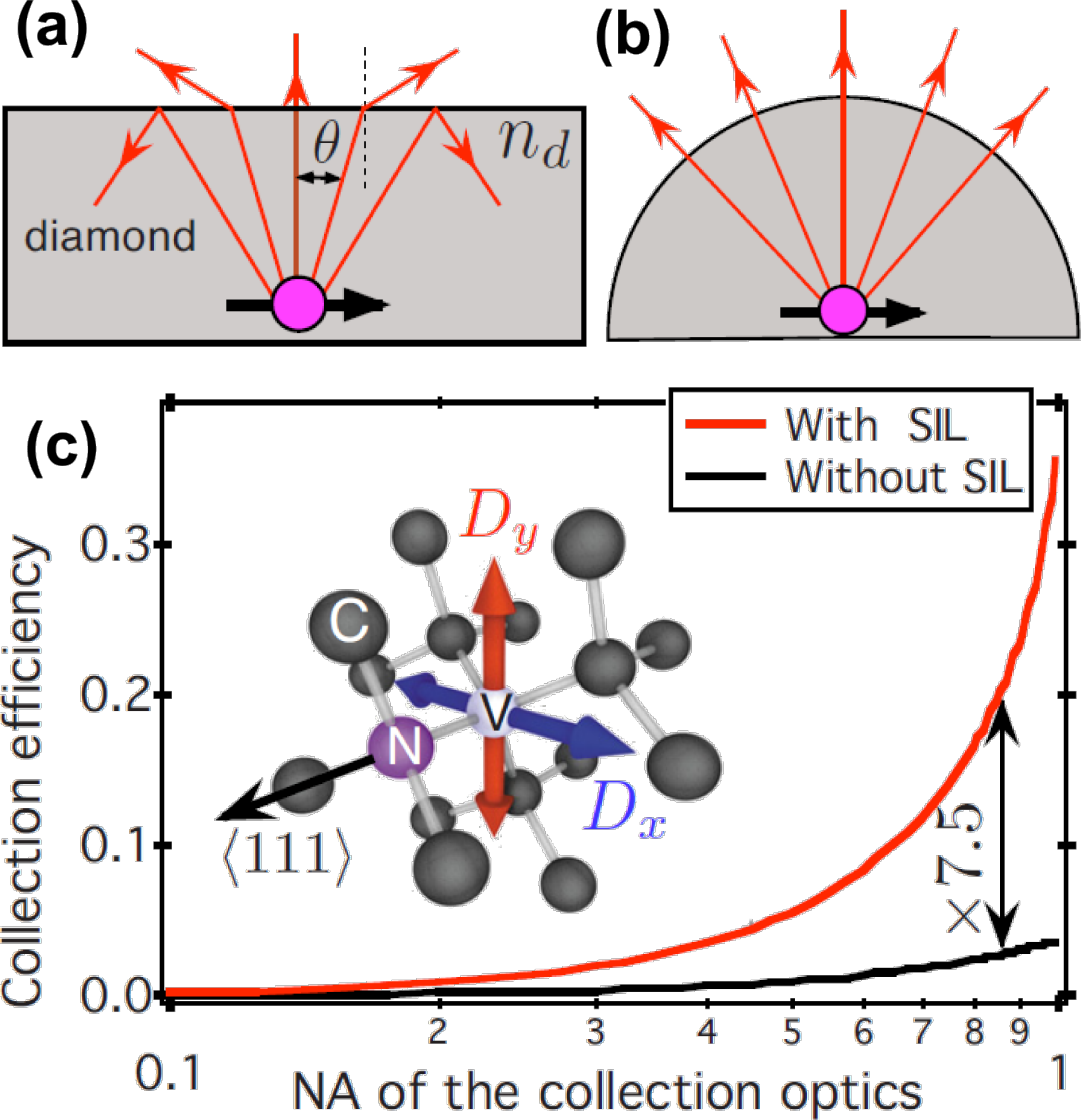
Enhancement of the collection efficiency with a hemispherical solid immersion lens (SIL). (a) Reduction of the effective numerical aperture due to refraction at the surface of a high-index medium. (b) Elimination of refraction with a hemispherical lens. (c) Collection efficiency as a function of numerical aperture with (red line) and without (black line) the solid immersion lens. Reproduced with permission from [10]. Copyright 2010 American Institute of Physics.

While plasmonic resonators focus on strong coupling between quantum emitters and resonators, frequently the most important aspect is to collect as much light as possible from a single solid-state quantum emitter. In case of diamond, this task is challenging due to the high refractive index (*n* = 2.4) of the host material. Total internal reflection at the sample surface prevents light from traversing the diamond/air interface, and many of the emitted photons are effectively trapped inside the diamond. Figure 5a illustrates this effect. Due to refraction, the effective numerical aperture (NA) for light collection is strongly reduced. A device that is suitable to overcome this effect is a so-called solid immersion lens (SIL), i.e., a hemispherical lens fabricated out of diamond. Due to the hemispherical shape, all light rays that emanate from the center of curvature are normal to the surface of the sphere, such that no refraction occurs (Figure 5b). With such a lens, we expect an increase of the collection efficiency by 6 to 8 times [10], depending on the numerical aperture of the collection objective. Figure 5c shows the relation between the collection efficiency and the numerical aperture. The effect is particularly pronounced with large numerical apertures (NA = 0.70–0.95), which are typically used for high efficiency light collection from solid-state quantum emitters. There are two complementary ways to fabricate a hemispherical lens out of diamond. On the one hand, one can produce a macroscopic (about a millimeter) size lens. On the other hand, one can use a focused ion beam (FIB) to fabricate a micrometer-sized hemisphere around a preselected diamond color center. In the following, both approaches are discussed. We first focus on a macroscopic solid immersion lens.

**Figure 6:**
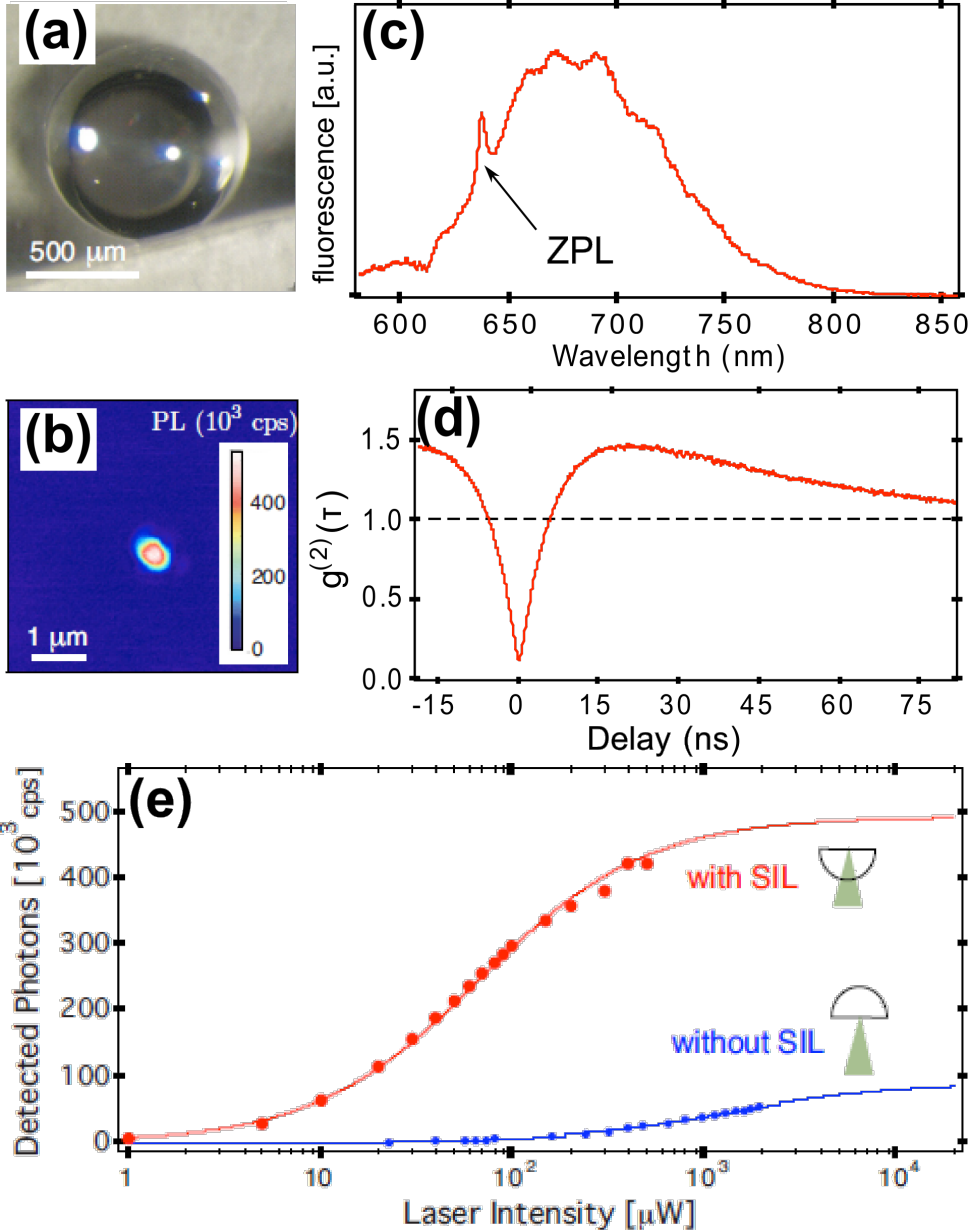
Macroscopic solid immersion lens [10]. (a) Photograph of a single crystalline diamond hemisphere. (b) Confocal fluorescence image of the focus plane. The bright spot in the center corresponds to a single nitrogen–vacancy defect. (c) Fluorescence emission spectrum. The peak at a wavelength of 637 nm (ZPL) corresponds to the resonant optical emission of the NV^−^ center. (d) Photon antibunching. The dip at time delay τ = 0 is well below 0.5, indicating a single quantum emitter. (e) Saturation curves with and without the solid immersion lens. Reproduced with permission from [10]. Copyright 2010 American Institute of Physics.

Figure 6a shows a photograph of a macroscopic hemispherical lens fabricated out of high-purity single-crystalline diamond (Element Six Ltd., London, UK). In this particular lens, a single color center is located close to the origin of the hemisphere and can be optically addressed. Figure 6b shows a confocal scanning microscope image of the focal region of the lens. The bright spot in the center of the image corresponds to a single color center. The nature of the color center can be determined by its fluorescence spectrum. Figure 6c shows the fluorescence spectrum. A characteristic resonant line at a wavelength of 637 nm (ZPL of NV^−^) is observed, identifying the color center as a negatively charged nitrogen–vacancy defect. To ensure that this is indeed a single quantum emitter, we measure its second-order photon autocorrelation. Figure 6d shows the data. The dip at time delay zero drops clearly below 0.5, showing that this is a single quantum emitter. Finally, we determine the maximum possible photon count rate achievable with the solid immersion lens. Figure 6e shows saturation curves of the single quantum emitter with and without taking advantage of the hemisphere. For this purpose, we probe the same quantum emitter, first through the flat bottom surface of the hemisphere and subsequently through the curved surface. The saturation curves are well described with a rate model for a two-level system. With this macroscopic diamond hemisphere, fluorescence count rates up to about 420 kHz are observed, which is sufficient for a number of important applications targeting low-temperature quantum control, such as single-shot electron-spin readout [11] or resonant-charge-state discrimination [12].

**Figure 7:**
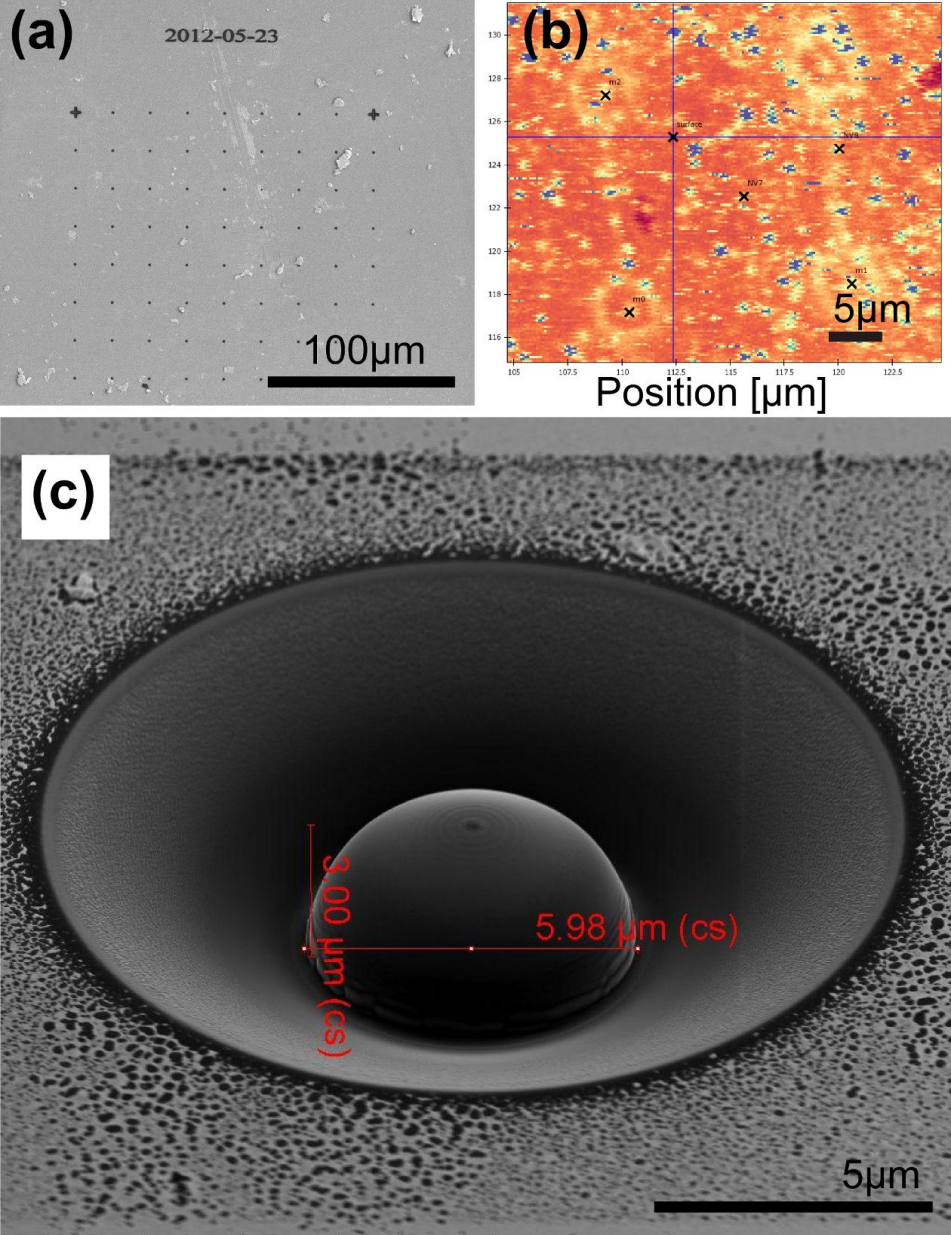
Fabrication of a microscopic diamond hemisphere by focused ion beam milling. (a) Grid of FIB markers for the precise alignment of the SIL on top of a single color center. (b) Fluorescence microscope image of one quadrant of FIB markers. The bright blue spots are single color centers. A color center a few microns below the surface (not visible in this image) is selected as the target emitter. Note that the image distortion between subsequent scan lines is caused by bi-directional motion of the imaging piezo scanner. (c) SEM image of a microscopic SIL. The complete FIB process takes approximately 30 min.

Due to the high cost associated with a macroscopic diamond lens, it would be very interesting to fabricate instead a microscopic hemisphere (ca. 10 micrometers) into the surface of a standard diamond sample. Due to the small size, thousands of microscopic lenses could be fabricated into a single diamond sample, and moreover, each lens could be fabricated precisely around a single fluorescent color center. A suitable fabrication technique is focused ion beam milling. We therefore explore whether this can be used to produce high-quality micrometer-sized diamond SILs. In order to register a SIL precisely on top of a single color center, we first fabricate a grid of markers (circular holes) into the surface of a diamond sample using a FIB. Figure 7a shows a scanning electron microscopy (SEM) image of such FIB markers. Subsequently, we analyze the sample with a confocal fluorescence microscope and determine single color centers within the grid (Figure 7b). The position of the color centers relative to the FIB markers as well as their depth below the diamond surface is determined with about 100 nm precision. Thereafter, a solid immersion lens with a radius corresponding to the depth of the color center is fabricated by using the focused ion beam. Figure 7c shows an example of such a microfabricated SIL. Note the cone around the SIL, which is fabricated in order not to cause additional refraction for the emitted fluorescence light. The ringlike ablation material at the bottom of the SIL lies within a spatial angle that is not detected by the high NA microscope objective of 0.95. With such a microscopic diamond hemisphere, fluorescence count rates up to about 480 kHz were observed, which is even slightly better than the result obtained with the macroscopic SIL. The device is therefore a highly promising microstructure that provides a universal performance boost for diamond quantum applications.

### 4 Dielectric pillar microcavities with embedded diamond nanocrystals

**Figure 8:**
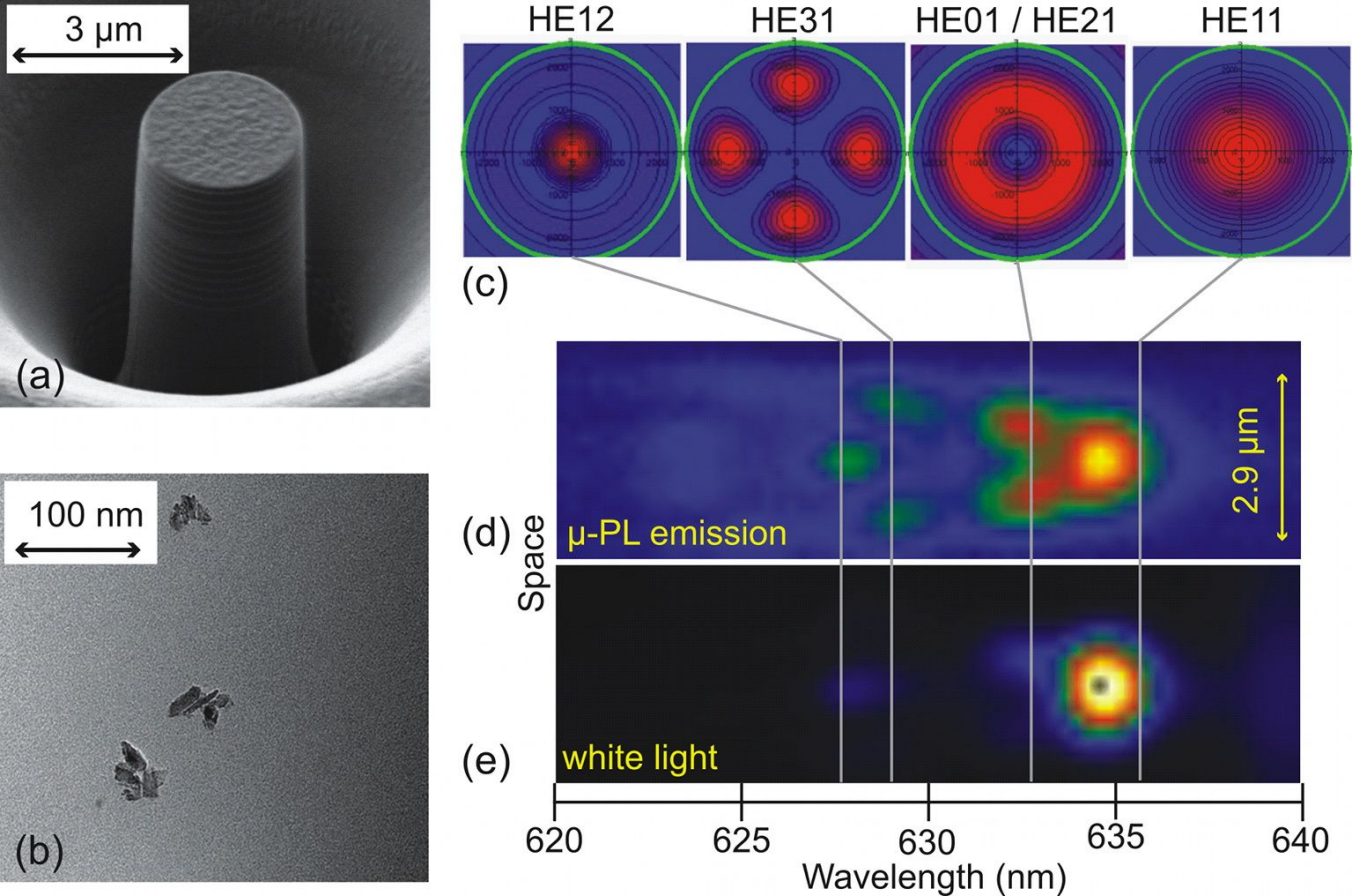
(a) Scanning electron microscopy (SEM) image of a micropillar resonator with embedded diamond nanocrystals in the central spacer layer. (b) Transmission electron microscopy (TEM) image of diamond nanocrystals. The average size of the nanodiamonds is 20 nm. (c–e) Mode spectrum of a pillar microcavity (2.9 µm diameter) with an ensemble of diamond nanocrystals in the spacer layer. (c) Simulated spatial cavity modes. (d) Spatio-spectrally resolved photoluminescence emission and (e) white-light transmission spectrum. Calculated spectral mode positions are indicated by vertical grey lines.

An alternative approach for increasing the collection efficiency exploits the Purcell effect. In this case, a single quantum emitter is placed into an optical resonator and the emitted photons exit preferentially into a resonator mode, much like the stimulated-emission process of a laser. The resonant photons can be coupled out with high efficiency from the resonator. In order to enhance the emission at the zero-phonon line (ZPL) of nitrogen–vacancy centers, diamond nanocrystals containing single NV centers were embedded into high quality pillar resonators (Figure 8a). In a first step, a bottom Bragg mirror composed of TiO_2_/SiO_2_ layer pairs is fabricated by magnetron radio-frequency sputtering. In a second step, nanodiamonds with a diameter of less than 20 nm (Figure 8b) are spin coated onto the dielectric mirror. The area density of the nanocrystals may be chosen by the concentration of the nanodiamond solution and/or by varying the rotation speed of the spin-coater. The nanocrystals are embedded in a TiO_2_ spacer layer, i.e., a region of a high index of refraction. Therefore, a so-called “λ-cavity” is manufactured. In a third step, the top Bragg mirror is sputtered to create a planar cavity structure with one-dimensional confinement of light. In order to achieve a three-dimensional light confinement, pillar microcavities are milled out of the planar structure by focused ion beam. As a consequence, the light field is concentrated vertically between the two dielectric Bragg mirrors and laterally due to the total internal reflection at the pillar sidewalls [13–14]. Due to the waveguide nature of pillar resonators, the photoluminescence emission is strongly directional, which results in the efficient collection of radiation with a microscope objective.

A broadband light transmission measurement through a single-pillar resonator is shown in Figure 8e. The transmission spectrum is dominated by the fundamental mode HE11 of the pillar microcavity. Also a faint first HE01/HE2 and third HE12 excited mode is discernible (Figure 8e). Since the incoherent light source generates an approximately planar wavefront, light preferably couples to the symmetric fundamental mode. The entire spectrum of cavity modes is clearly visible in the photoluminescence emission with continuous-wave laser excitation of an ensemble of diamond nanocrystals at a wavelength of 532 nm (Figure 8d). The spectral positions of these resonances (Figure 8d) are calculated based on an effective-waveguide model. The theoretical results are in excellent agreement with the values obtained by the experiment. The simulations also yield the spatial mode patterns depicted in Figure 8c. The recorded CCD image of the photoluminescence emission provides us with one-dimensional spatial resolution along the entrance slit of the spectrometer. For this reason a vertical cut through the center of the calculated two-dimensional mode patterns (Figure 8c) can be compared to the CCD image in Figure 8d. In particular, the fundamental mode HE11 exhibits one intensity maximum. The first excited mode HE01/HE21 has two constituents.

**Figure 9:**
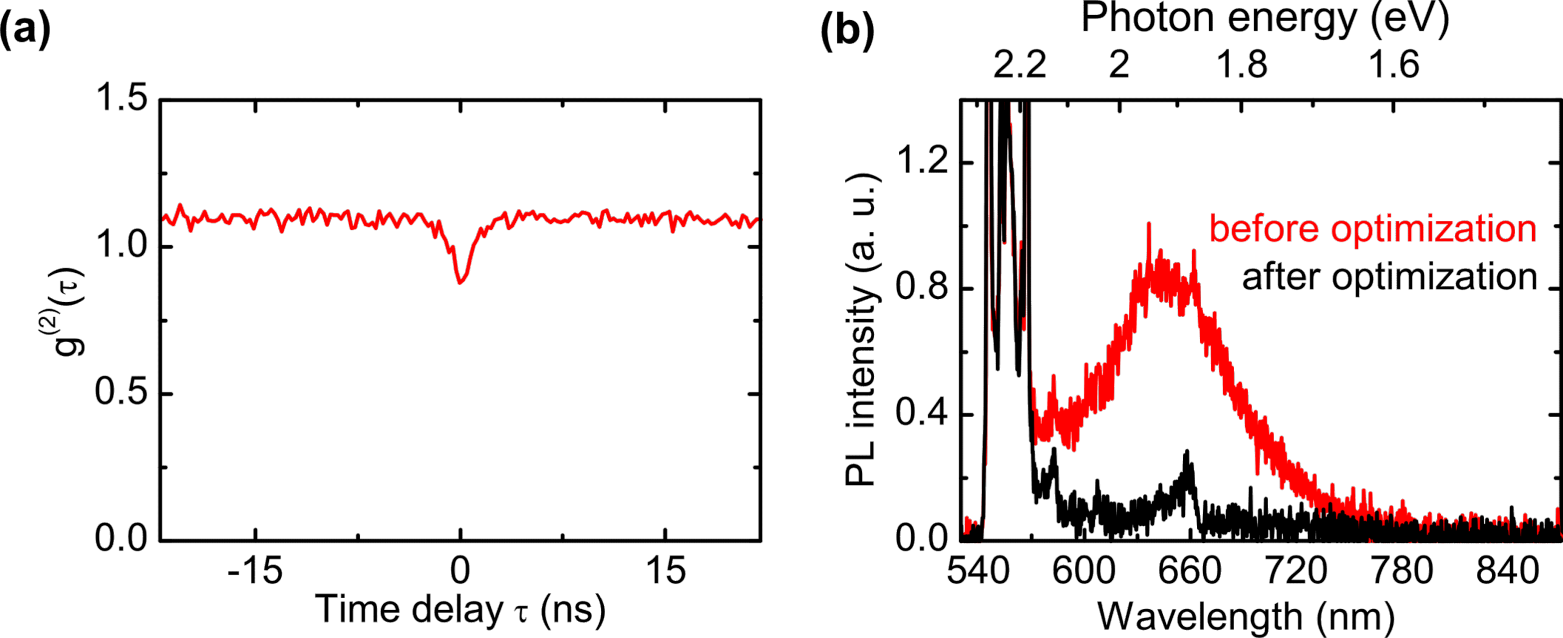
(a) Normalized intensity autocorrelation function g^(2)^(τ) from a micropillar cavity of 1.6 µm diameter with embedded diamond nanocrystals. (b) Comparison between the photoluminescence emission from a standard (black) and an optimized (red) sputtered SiO_2_ layer.

To elucidate the nature of photon emission and characterize the coupling of NV centers to an optical cavity, we perform second-order autocorrelation measurements. The normalized intensity function g^(2)^(τ) is recorded with an optical setup according to Hanbury Brown and Twiss. As seen in Figure 9a, a clear antibunching effect (g^(2)^(τ) < 1) can be observed at zero time delay (τ = 0) from an optical cavity with 1.6 µm diameter. This is strong evidence for nonclassical light emission. The depth of the antibunching dip at zero time delay amounts to 0.21. A value below 0.5 would explicitly indicate that a single NV center coupled to the cavity is a single photon source. There are two main factors that may currently limit the depth of the antibunching dip. First, background photoluminescence from the dielectric materials, especially from SiO_2_ layers, and second, background emission from the diamond nanocrystal itself. By optimization of the sputter parameters, we recently achieved a reduction in photoluminescence of sputtered SiO_2_ layers by a factor of nine at a wavelength of 637 nm (ZPL of NV^−^ centers, Figure 9b). In the future, this process optimization may be used to reduce the background photoluminescence of the cavity.

### 5 Fabrication of Ni-, W- and Si-based color centers in CVD diamond

#### 5.1 Motivation

Diamond is an excellent host for fluorescent defects. Due to the large band-gap energy of 5.46 eV, it is likely that localized defect states are present in the forbidden band. Not surprisingly over 500 such “deep trap” centers are meanwhile known in diamond [15–16]. Their fluorescence emission covers a broad spectral range reaching from the UV to the far IR. Admittedly, origin and composition of many color centers in diamond are yet not well understood or even known. Above all, not every fluorescent defect center exhibits the desired characteristics for applications in quantum information processing [17], such as a small bandwidth, a low electron–phonon coupling, or a high oscillator strength. For this reason two main challenges need to be addressed by diamond researchers:

- New color centers in diamond with favorable properties for quantum-information-processing technologies need to be identified.
- A reproducible fabrication method for color centers (such as the nickel-related NE8-center) in high-quality diamond layers has to be developed.

As demonstrated and discussed in Section 1, ion implantation into bulk diamond crystals of high purity is a well-established technique [4] to produce defects such as the NV center, offering nanometer spatial resolution and a controlled defect density. A drawback, on the other hand, is the inevitable damage caused to the diamond lattice, which leads to an additional photoluminescence (PL) background and disturbances of the photoluminescence emission [18]. Furthermore, not every color center in diamond can be produced by implantation with an adequate yield [15]. The direct synthesis of diamond crystals by microwave plasma enhanced chemical vapor deposition (MWPECVD) offers an alternative route to integrate color centers and was investigated in addition to ion implantation [19–20]. We deposited single-crystal diamond layers of high phase and structural purity by MWPECVD. Emphasis was placed on a reproducible dopant addition to the growth process aiming at a targeted in situ incorporation of color centers based on nickel and tungsten impurities.

A very promising single-photon-emitting defect for quantum-cryptographic applications is the so-called NE8-center [17]. This nickel–nitrogen based defect exhibits superior properties, such as a sharp emission line at around 800 nm, with a width of only 2 nm at room temperature, together with a short intrinsic lifetime of 2 ns, and an efficient emission concentrated in the zero-phonon line. Different attempts were already conducted to produce this center [21–22]. However, the yield of nickel–nitrogen-related centers seems to be rather low. Tungsten is known to produce a family of so-called W_5_-centers with several luminescence lines near 714 nm [23]. Up to now these centers were only produced by chance in polycrystalline diamond samples grown by the hot-filament technique. Accordingly, not much is known about their luminescence properties. Our aim was to produce the W-centers in a well-defined way, in order to enable further studies on these color centers.

The goal of our work is the fabrication of stable single-photon emitters with a high emission rate in the red and infrared spectral range. However, as discussed in Section 3 the high refractive index of diamond impedes light extraction from the bulk, thereby lowering the achievable count rates. A further way to circumvent this disadvantage is to implement color centers in small nanodiamond crystals with diameters well below the wavelength, guaranteeing an efficient light extraction. Moreover, single nanodiamonds can be implemented into dielectric cavities enhancing the efficiency, as demonstrated in Section 4. We will discuss our approaches to incorporate silicon–vacancy (SiV)-centers in dispersed nanodiamond particles fabricated by MWPECVD in Section 5.5.

#### 5.2 Nickel and tungsten doping of single-crystal diamond layers

**Figure 10:**
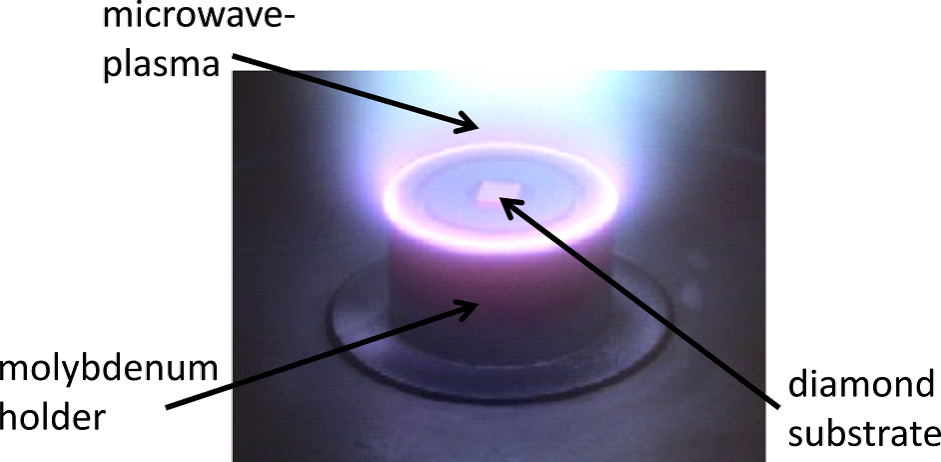
View into the MWPECVD reactor during growth of a single-crystalline diamond layer.

**Homoepitaxial growth of diamond** - Homoepitaxial diamond growth was performed at low pressure conditions in a microwave-activated hydrogen-rich plasma atmosphere in an ellipsoidal cavity reactor [24]. The necessary carbon species for the diamond growth were supplied by the addition of 1–2% methane to the process gas. Single-crystal diamond plates of type Ib with (001) and (111) surface orientation and dimensions of 3 × 3 mm² served as substrates. In order to provide optimal growth conditions, a high plasma-power-density regime (100–150 W/cm³) was achieved by applying a pressure of 200 mbar and a microwave power of 2–3 kW. Figure 10 shows the holder configuration we used to focus the plasma ball. The main advantage of such a small reaction volume is that neither the reactor base plate nor the quartz walls of the surrounding bell jar is touched by the plasma. Major contaminations from the reactor walls were therefore avoided.

**Gaseous nickel and tungsten precursors** - The controlled and targeted addition of dopants during diamond growth is a crucial step for the in situ synthesis of color centers. A frequently applied method is to expose a solid state source containing the dopant material directly to the reactive plasma [15,19,21]. We also used this approach for the doping of nanodiamond crystals with silicon, which will be discussed in section 5.5. A drawback of a solid-state doping source is the limited control over the dopant concentration during growth. To ensure a reproducible doping we studied the applicability of gaseous metal precursors, namely nickelocene Ni(C_5_H_5_)_2_ and tungsten hexacarbonyl W(CO)_6_, for the doping of diamond with nickel and tungsten. Both precursors are solids at room temperature but exhibit a high vapor pressure [20]. Nickelocene and tungsten hexacarbonyl, separately, were sublimated in a temperature-controlled dopant reservoir. Argon was passed through this reservoir, saturated with the vapor of one of the precursors and afterwards introduced to the process chamber. By using an inert carrier gas, we paid attention to the fact that the two precursors are unstable in a hydrogen-rich atmosphere [20]. We thereby avoided a premature decomposition of the precursors in the gas line.

**Figure 11:**
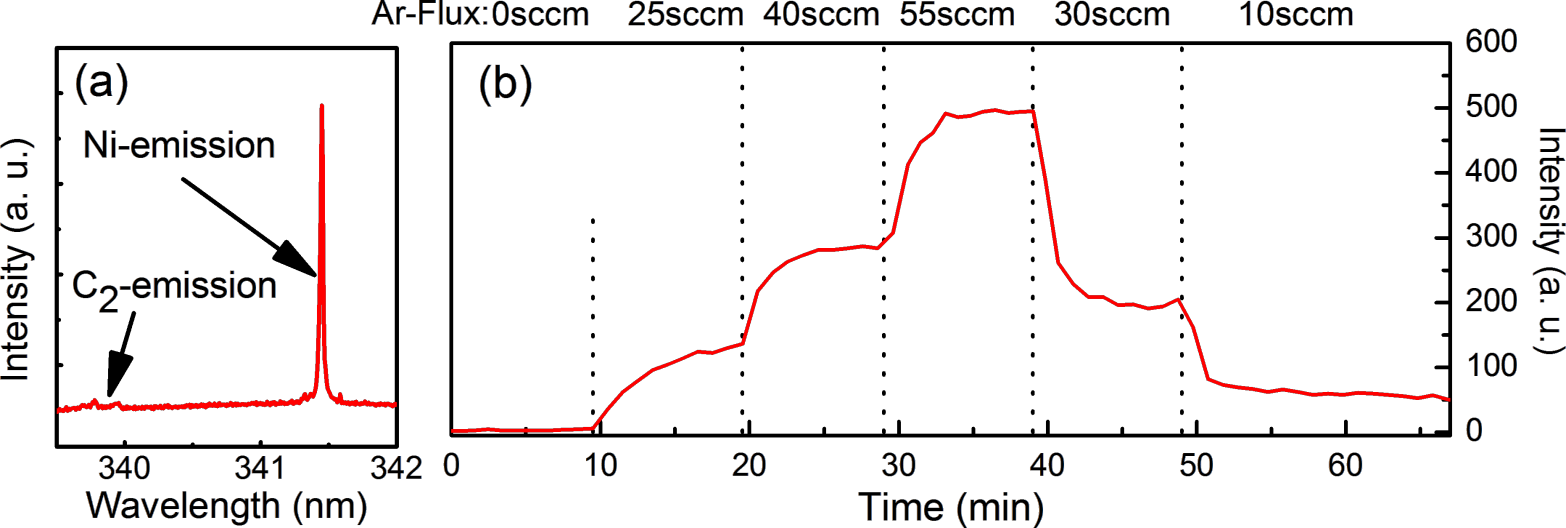
**(**a) Optical emission spectroscopy: observed nickel emission in the MWPECVD plasma during diamond growth with addition of nickelocene. (b) Temporal evolution of the nickel emission (341.47 nm line) during MWPECVD diamond growth altering the argon/nickelocene addition. The intensity of the nickel emission is solely determined by the carrier gas flux to the reactor. Furthermore, the nickel emission is steady for constant nickelocene additions thereby demonstrating the reproducibility of the utilized gas-phase-doping approach. Reproduced with permission of the author from [25].

A major advantage of our gas-phase-doping approach is that it offers a targeted dopant addition. The dopant concentration in the gas phase can be adjusted either by the temperature of the precursor material in the dopant reservoir or by the carrier gas flux. The reproducibility of our doping approach was verified by checking for nickel- or tungsten-related emission lines in the MWPECVD plasma by using optical emission spectroscopy (OES). Tungsten transitions in the MWPECVD plasma are rather weak and it was difficult to separate them from the bright plasma background. Nickel exhibits in contrast some prominent emission lines in the UV. We choose an intense transition with an emission line at a wavelength of 341.47 nm, as shown in Figure 11a, to study the addition of nickelocene. Figure 11b shows the temporal evolution of the 341.47 nm emission for different argon fluxes through the nickelocene bubbler. During the first 10 min no nickelocene was added to the carrier gas. Accordingly, no nickel signal is visible. Afterwards the carrier gas flux was altered every 10 min. The signal presented in Figure 11b follows this variation. Furthermore, a steady signal is visible for a stable argon addition. The rapid decrease of the nickel signal after lowering the argon flux at both 40 min and 50 min indicates the absence of unwanted hysteresis effects.

#### 5.3 Verification of nickel incorporation

**Figure 12:**
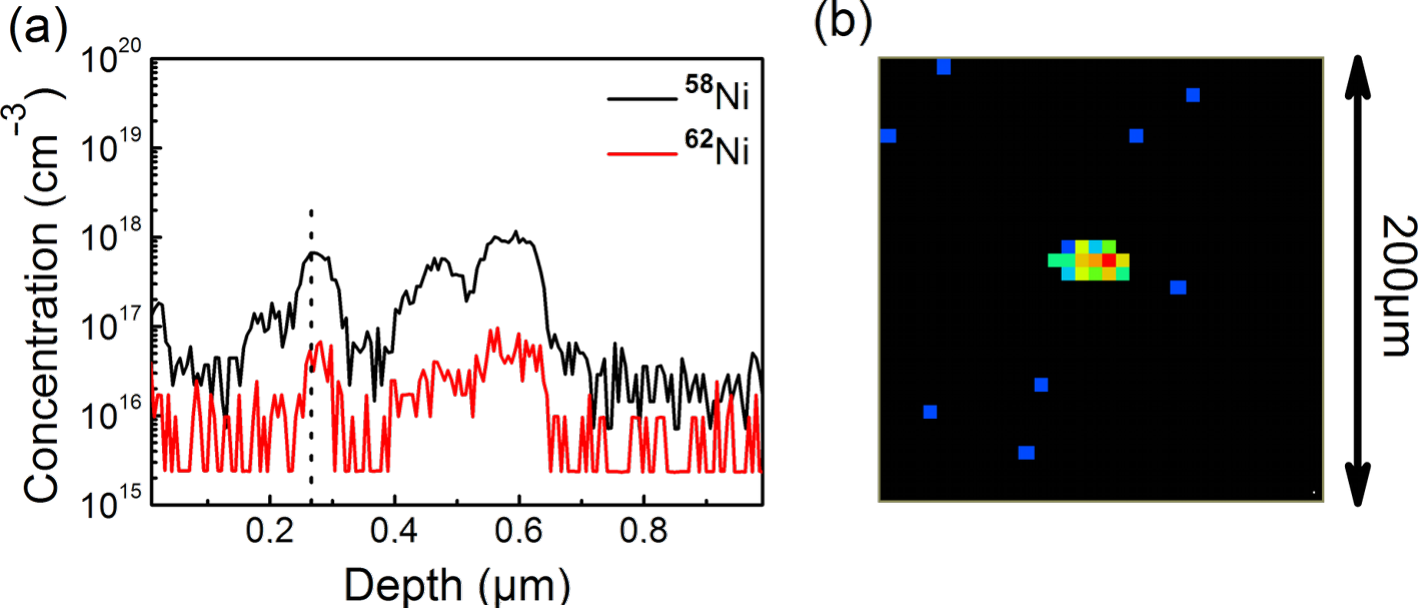
(a) SIMS depth profile of nickel-doped single-crystal diamond layer. The intensity of the two “marker” isotopes ^58^Ni and ^62^Ni is in accordance with the known natural abundance, thereby verifying nickel incorporation. (b) SIMS-signal from picture (a) observed at a depth of 0.25 µm. The nickel signal emanates from a spot indicating the formation of nickel clusters during diamond growth with simultaneous nickelocene addition. Reproduced with permission of the author from [25].

The incorporation of nickel into the as-grown diamond layers was verified by secondary ion mass spectrometry (SIMS). An unambiguous assignment of nickel was achieved by measuring two different nickel isotopes (namely ^58^Ni and ^62^Ni) and comparing the measured count rates with the known natural abundances of these isotopes. Figure 12(a) shows a SIMS measurement performed on a 500 ± 100 nm thick diamond layer grown with constant nickelocene addition. During MWPECVD growth a mole fraction of 10^−7^ of nickelocene was added to the process gas. The SIMS measurement reveals that nickel concentrations up to 10^18^ cm^−3^ are present in this layer. However, although it was verified by OES that a constant amount of nickelocene was added during the deposition process, a nonuniform depth profile is visible. Furthermore, the nickel signal was not uniformly distributed in the lateral direction, as seen in Figure 12b. Both results indicate that nickel clusters were formed during growth, and nickel is rather encapsulated than incorporated into the diamond film.

**Figure 13:**
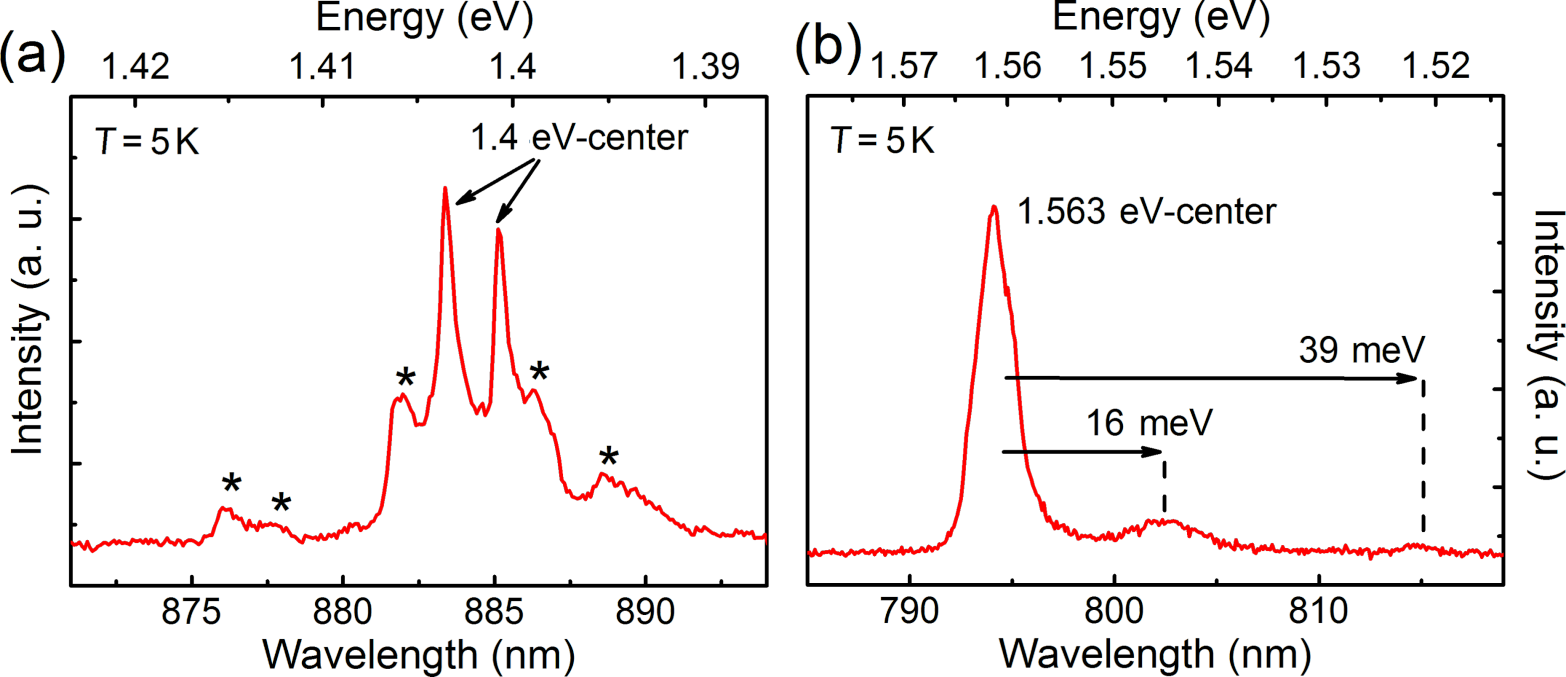
Cathodoluminescence spectra measured at a temperature of 5 K on a nickel-doped single-crystal diamond layer. (a) Emission lines of the 1.4 eV center at 883.4 nm and 885.1 nm, verifying nickel incorporation. Origins of accompanying lines marked with an asterisk are unknown. (b) Luminescence of the 1.563 eV center (“NE8-defect”) observed in the same diamond layer. Reproduced with permission of the author from [25].

Additional measurements were performed to ensure the incorporation of nickel atoms into the diamond lattice. Of avail in this context is that nickel is known to form the so-called 1.4 eV defect in diamond [16]. This fluorescent center exhibits two narrow lines at a wavelength of around 884 nm, which are readily excited by cathodoluminescence (CL). Figure 13 shows a CL-measurement performed on a nickel-doped diamond layer. Several lines at a wavelength of around 884 nm are visible in the spectra of the nickel-doped layer. The two most prominent lines are located at 883.37 nm (1.4035 eV) and 885.12 nm (1.4008 eV), which is in accordance with values known for the 1.4 eV center [16].

CL measurements on the same nickel-doped sample revealed further nickel-related lines. As shown in Figure 13b a luminescence line at a wavelength of 794 nm was detected accompanied by two phonon sidebands on the lower energy side, shifted by 16 meV and 39 meV. The origin of this luminescence line is the 1.563 eV center, also sometimes referred to as the “NE8-center”. From this observation it can be concluded that nickel was not solely encapsulated during MWPECVD growth. In fact nickel-related color centers were produced by using nickelocene as a nickel precursor during diamond MWPECVD growth.

#### 5.4 Verification of tungsten incorporation

**Figure 14:**
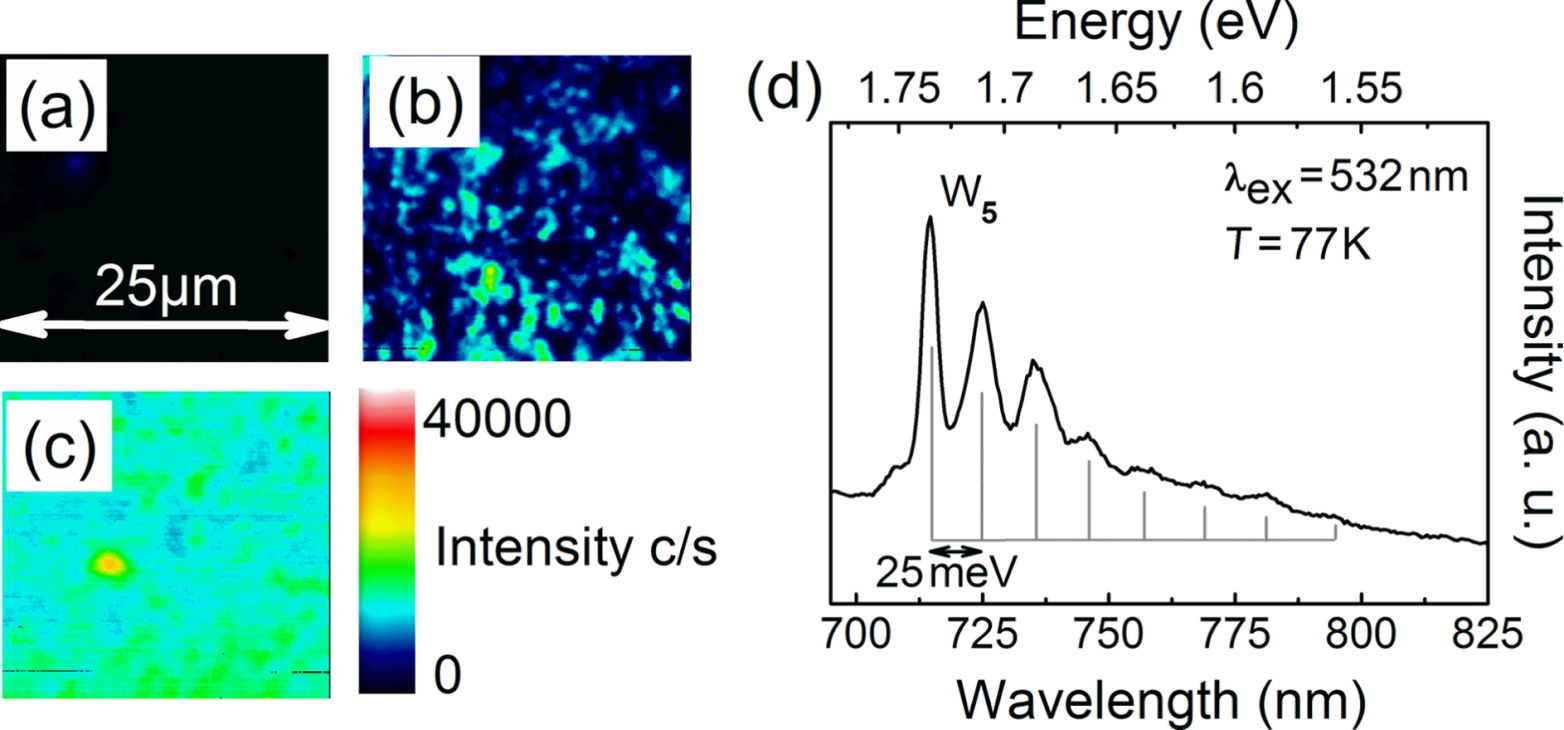
(a–c) Room-temperature PL mapping excited at a wavelength of 660 nm on (111) diamond layers grown by MWPECVD with different additions of W(CO)_6_: (a) no addition (reference layer); (b) with a mole fraction of 1.1 × 10^−6^ W(CO)_6_ in the process gas; (c) with a mole fraction of 1 × 10^−5^ W(CO)_6_ in the process gas. (d) Photoluminescence spectrum obtained from a tungsten-doped single-crystal diamond layer; acquired at 77 K with 532 nm laser excitation. The tungsten-related W_5_-luminescence at a wavelength of 714 nm together with several pronounced phonon sidebands is visible. Reproduced with permission of the author from [25].

A direct verification of tungsten incorporation into the as-grown diamond layers by SIMS was not possible, thus indicating that the tungsten concentrations were well below the detection limits of 10^16^–10^17^ cm^−3^. However, confocal micro-photoluminescence measurements provided confirmation of tungsten incorporation. Layers grown with the addition of W(CO)_6_ exhibited a broad luminescence with emission in the spectral window between 680 nm and 825 nm. Additional features became visible when PL measurements were performed at a temperature of 77 K as shown in Figure 14d. The bright line at a wavelength of 714 nm is the zero-phonon line of the emission. The ZPL is accompanied on the lower energy side by several phonon sidebands, which are nearly equidistantly spaced by 25 meV. Position of the ZPL, as well as of the sidebands, are in agreement with the so-called W_5_-center [16,23]. The same luminescence emission was reported in the past for diamond layers deposited by the hot-filament technique and accordingly by the DC-arcjet technique [16,23]. The W_5_-center was hence ascribed to impurities originating from the filament material and accordingly from the cathode material of the reactor systems. The observation of the W_5_-luminescence in diamond layers grown with the addition of W(CO)_6_ confirms the previous assignment.

The gas-phase doping approach offered furthermore the possibility to adjust the density of W_5_ centers by the addition of W(CO)_6_ during diamond deposition. The µ-PL mappings on samples doped with different additions of W(CO)_6_ in Figure 14a–c confirm this statement. The tungsten concentration during growth was raised by a factor of ten going from Figure 14b to Figure 14c. The intensity of the W_5_-luminescence increases accordingly. Moreover, no dark areas without W_5_-center luminescence are visible in Figure 14c.

#### 5.5 Silicon–vacancy centers in as-grown nanodiamonds

**Figure 15:**
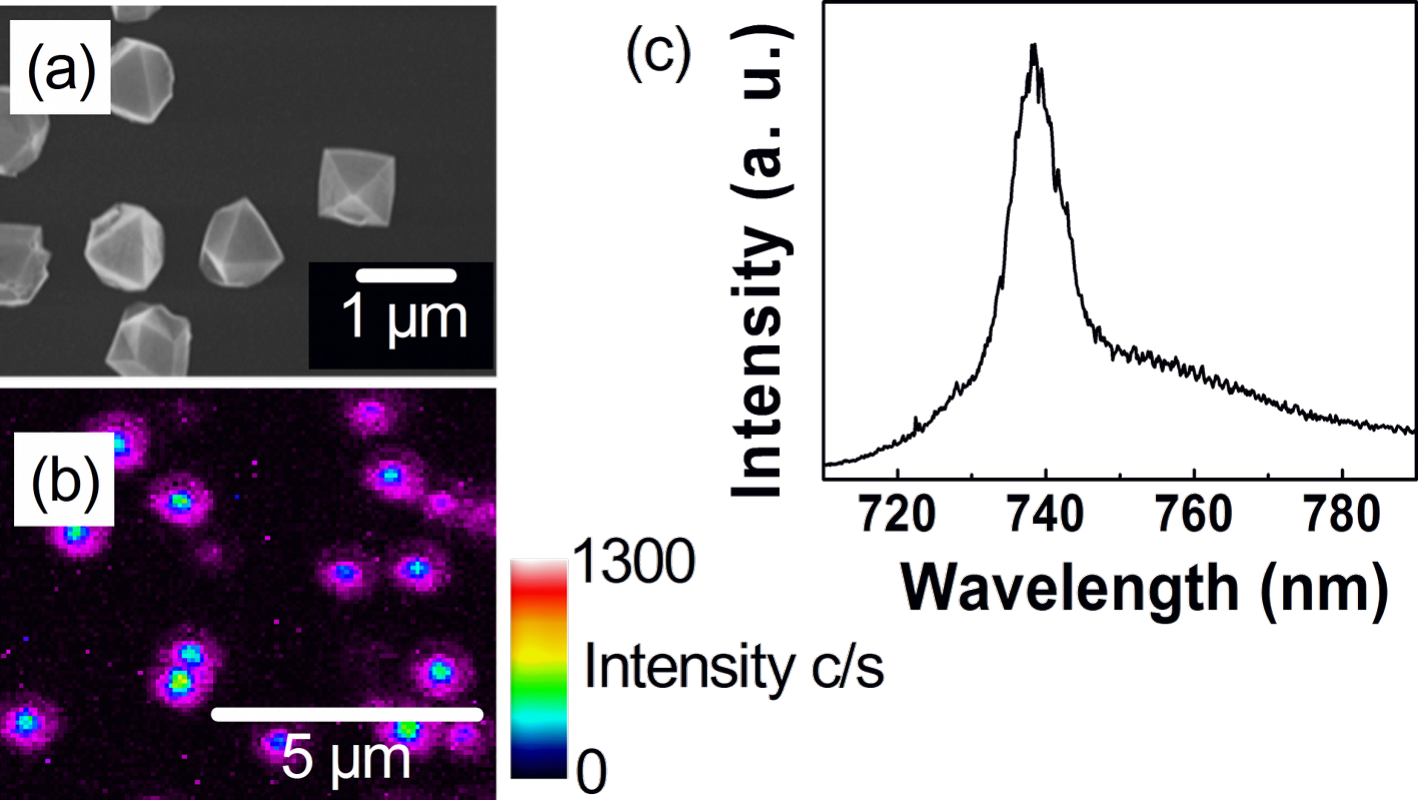
(a) As-grown nanodiamond particles on a silicon substrate. (b) Confocal photoluminescence mapping (660 nm excitation wavelength), recorded at room temperature. The integrated intensity emitted in the spectral window between 725 and 755 nm was measured. Bright areas in the scan correlate with nanodiamond particles. (c) Spectrally resolved PL emission from a particle in (b). The SiV luminescence verifies the successful incorporation of silicon during growth.

**Seeding and overgrowth of dispersed nanodiamonds on silicon** - We seeded nanodiamond particles with diameters below 10 nm from a colloidal solution onto a silicon wafer [26]. By controlling the surface chemistry of the particles it was possible to achieve an average distance of ca. 1 µm between two adjacent particles. The low particle density guaranteed that every particle could be addressed individually afterwards by confocal microscopy [27]. The nanodiamonds acted as seed crystals in the subsequent overgrowth in the MWPECVD plasma process. In this manner, we produced particles with diameters up to 700 nm, as shown in Figure 15a. Because of the harsh plasma environment the silicon substrate is slightly etched and the plasma is enriched with silicon. Silicon atoms are therefore also incorporated into the growing nanodiamond particles.

**Photoluminescence characterization** - The presence of silicon-related color centers in the as-grown nanodiamond particles was investigated by micro-photoluminescence measurements. Figure 15b shows a typical PL mapping over a 20 × 20 µm² region. The bright areas correlate with nanodiamond particles. Origin of the PL signal is the luminescence of the SiV center. A typical PL spectrum of the SiV center is shown in Figure 15c. In order to enhance the yield of fluorescent silicon defects, we varied the temperature during the MWPCVD growth process. Diamond growth below 600 °C is comparatively slow. However, even small nanodiamonds exhibit SiV luminescence. Nanodiamonds grown above 600 °C exhibited a lower SiV yield. Especially small particles showed no luminescence.

## Conclusion

We have performed a series of key experiments towards strong coupling of solid-state quantum emitters to plasmonic and dielectric optical resonators. First, we have demonstrated controlled creation of nitrogen–vacancy centers in diamond with nanometer spatial control by ion implantation through suitable masks. Using high-aspect-ratio mica masks, we have shown that high-energy deep implants can be created with nanometer spatial precision. Subsequently, we have demonstrated the coupling of diamond nanocrystals to plasmonic resonators. Emitter–resonator coupling was achieved for different aluminium resonator geometries, with a shortening of the excited state lifetime six times. By using dielectric diamond hemispheres, the photon collection efficiency was increased by a factor of up to six. Dielectric pillar microcavities with embedded diamond nanocrystals containing single NV centers have been manufactured by sputtering and focused ion beam milling. Photon antibunching from a NV center inside a dielectric pillar cavity could be observed. In addition, a reproducible gas-phase doping approach to incorporate nickel and tungsten atoms during MWPECVD growth of single-crystal diamond films has been demonstrated. Our experiments mark a firm step towards strong coupling of solid-state quantum emitters to plasmonic and dielectric resonators by using integrated plasmonic nanophotonics and dielectric diamond optics.
